# Dosing Optimization of Posaconazole in Lung-Transplant Recipients Based on Population Pharmacokinetic Model

**DOI:** 10.3390/antibiotics12091399

**Published:** 2023-09-01

**Authors:** Eliška Dvořáčková, Martin Šíma, Andrea Zajacová, Kristýna Vyskočilová, Tereza Kotowski, Kateřina Dunovská, Eva Klapková, Jan Havlín, Robert Lischke, Ondřej Slanař

**Affiliations:** 1 Department of Pharmacology, First Faculty of Medicine, Charles University and General University Hospital in Prague, 128 00 Prague, Czech Republic; eliskadvorackova@seznam.cz (E.D.); ondrej.slanar@lf1.cuni.cz (O.S.); 2 Prague Lung Transplant Program, Department of Pneumology, Second Faculty of Medicine, Charles University in Prague and Motol University Hospital, 150 06 Prague, Czech Republic; andrea.zajacova@fnmotol.cz (A.Z.); kristyna.vyskocilova@fnmotol.cz (K.V.); tereza.kotowski@fnmotol.cz (T.K.); 3 Department of Medical Chemistry and Clinical Biochemistry, Second Faculty of Medicine, Charles University in Prague and Motol University Hospital, 150 06 Prague, Czech Republic; katerina.dunovska@fnmotol.cz (K.D.); eva.klapkova@fnmotol.cz (E.K.); 4 Prague Lung Transplant Program, 3rd Department of Surgery, First Faculty of Medicine, Charles University in Prague and Motol University Hospital, 150 06 Prague, Czech Republic; jan.havlin@fnmotol.cz (J.H.); robert.lischke@fnmotol.cz (R.L.)

**Keywords:** posaconazole, antimycotics, lung transplantation, therapeutic drug monitoring, nonlinear mixed-effects model, covariates

## Abstract

Although posaconazole tablets show relatively low variability in pharmacokinetics (PK), the proportion of patients achieving the PK/PD target at the approved uniform dose for both prophylaxis and therapy is not satisfactory. The aim of this study was to develop a posaconazole population PK model in lung-transplant recipients and to propose a covariate-based dosing optimization for both prophylaxis and therapy. In this prospective study, 80 posaconazole concentrations obtained from 32 lung-transplant patients during therapeutic drug monitoring were analyzed using nonlinear mixed-effects modelling, and a Monte Carlo simulation was used to describe the theoretical distribution of posaconazole PK profiles at various dosing regimens. A one-compartment model with both linear absorption and elimination best fit the concentration–time data. The population apparent volume of distribution was 386.4 L, while an apparent clearance of 8.8 L/h decreased by 0.009 L/h with each year of the patient’s age. Based on the covariate model, a dosing regimen of 200 mg/day for prophylaxis in patients ˃60 years, 300 mg/day for prophylaxis in patients ˂60 years and for therapy in patients ˃60 years, and 400 mg/day for therapy in patients ˂60 years has been proposed. At this dosing regimen, the PK/PD target for prophylaxis and therapy is reached in 95% and 90% of population, respectively, representing significantly improved outcomes in comparison with the uniform dose.

## 1. Introduction

Lung transplant recipients are at significant risk of invasive fungal infections, with a reported cumulative incidence of 8.6% during the first year after transplantation [1]. Invasive fungal diseases are the second leading cause of death, affecting 10% of lung transplant recipients [2]. Therefore, the antifungal prophylaxis plays a crucial role in post-operative management [3]. The guidelines of the International Society for Heart and Lung Transplantation (ISHLT), the American Society of Transplantation Infectious Diseases Community of Practice (AST), and the Infectious Diseases Society of America (IDSA) recommend post-transplant prophylaxis with a spectrum inclusive of *Aspergillus* spp. [4,5,6]. However, there has been no consensus on the strategy (targeted vs. universal) and preferred agent (inhaled amphotericin B, systemic azole agents, or their combination). The guidelines recommend an extended period of prophylaxis (3–6 months) in high-risk lung-transplanted patients, and in patients who develop an invasive infection, the duration of therapy can range from 6 weeks to a lifetime. Although there is a great heterogeneity in the choice of agents among lung-transplant centers, posaconazole is the most commonly used medication [7].

Posaconazole shows formulation-dependent pharmacokinetics (PK). Currently, it is available in two formulations for oral administration: tablets and suspension. The utilization of suspension is limited due to low and highly variable posaconazole bioavailability, which is dependent on food, gastric acidity, and intestinal motility [8]. In comparison, tablets show better bioavailability that is independent of pH and motility. Tablets can be administered in one daily dose and only high-fat meals can increase the exposure to the posaconazole administered [9]. Posaconazole is lipophilic, demonstrates a large volume of distribution (Vd) of 294–583 L, and is highly bound to plasma proteins (>98%) [9]. The parent drug is predominantly metabolized via hepatic glucuronidation, and both the parent drug and metabolites are subsequently excreted in feces (major part) and urine (minor part) with an elimination half-life (t_1/2_) of 26–31 h [9]. The Summary of Product Characteristics (SmPC) recommends an approved uniform dose of 300 mg once daily for all patients for both prophylaxis and therapy. Nevertheless, several factors affecting the variability of posaconazole serum levels have been described, e.g., race, age, gender, liver function test levels, or episodes of gastrointestinal disturbances [10]. It should also be emphasized that different PK/PD targets are defined for prophylaxis and therapy. In vitro experiments and animal models have shown that for posaconazole, similarly as for other azole antifungals, the area under the concentration–time curve over 24 h divided by the minimal inhibitory concentration (AUC/MIC ratio) is the PK/PD index that is most associated with efficacy [11]. The AUC/MIC ratios approaching a value of 200 have been found to be predictive of the successful treatment of *Aspergillus* spp. [8]. Given the difficulty of determining the AUC under routine clinical practice and the tight correlation between total posaconazole exposure and its trough concentrations [12], the monitoring of posaconazole trough levels is recommended as a PK/PD target in clinical practice [8,13]. Clinical studies suggest trough levels of >0.7 mg/L for prophylaxis and trough levels of >1.25 mg/L for the treatment of invasive fungal diseases as targets, minimizing the risk of breakthrough of invasive fungal infections [8,11]. These breakpoints are also recommended by the European Committee on Antimicrobial Susceptibility Testing (EUCAST) [13].

A retrospective analysis among patients of the Munich Lung Transplantation Program showed that at the approved dosing, only 82% and 76% of posaconazole trough levels measured during therapy and prophylaxis, respectively, meet the above-mentioned targets [14].

Several population PK models have been proposed for posaconazole in the past [10]. However, to our knowledge, a population PK model of posaconazole in lung-transplant patients has not yet been developed and subsequently used for dosing optimization.

Therefore, the aim of this study was to analyze posaconazole therapeutic drug monitoring (TDM) data from lung-transplant recipients using nonlinear mixed-effects modeling and then to propose covariate model-based dosing optimization for both prophylaxis and therapy in order to improve the proportion of patients achieving the PK/PD target.

## 2. Results

### 2.1. Study Population

There were 32 patients (10 females, 22 males) enrolled in this study. Their demographic and laboratory characteristics at the beginning of therapy are summarized in Table 1. Lung transplantation was indicated for cystic fibrosis, idiopathic pulmonary fibrosis or other fibrotic changes, interstitial lung disease, chronic obstructive pulmonary disease, bronchial asthma, and chronic aspergillosis in 3, 7, 13, 7, 1, and 1 patients, respectively. All patients received prophylaxis of acute organ rejection with basiliximab or antithymocyte globulin, based on their immunological risk. All patients also received immunosuppression. There were thirty-one patients treated with tacrolimus, and one patient received cyclosporin A. In addition, all patients received prednisone and 31 patients were treated with mycophenolate mofetil. Drugs increasing gastric pH (proton pump inhibitors or famotidine) were used by 29 patients.

The initial dosage of posaconazole was 300 mg once daily and was subsequently adjusted during treatment according to the measured levels (100–400 mg once daily). A total of 80 measured posaconazole serum levels were included in PK analysis (1–12 per patient, 2.5 per patient in average, modus two sampling points per patient).

### 2.2. Pharmacokinetic Analysis

**Figure 1:**
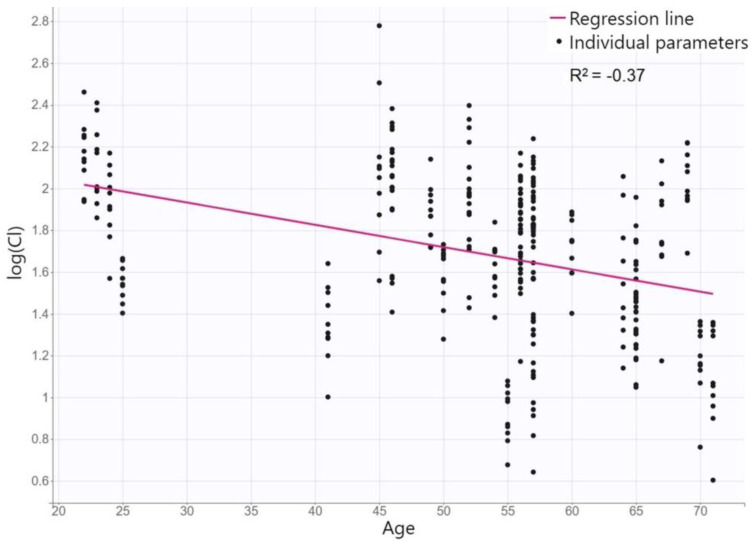
Covariate model diagnosis: relationship between conditional distribution of individual apparent posaconazole clearance and age.

For describing the posaconazole concentration–time data and residual and interpatient variability, a one-compartment structural model with both linear absorption and elimination kinetics and with a proportional error was the most appropriate. The population estimates of the posaconazole PK parameters in the final model are summarized in Table 2. Based on a covariate model diagnosis among all tested variables, the only one significant covariate was age for CL/F (see Figure 1), where posaconazole population CL/F of 8.8 L/h decreases by 0.009 L/h with each year of the patient’s age. The *p*-values for relationships of the potential covariates tested and the posaconazole PK parameters are summarized in Table 3. The final equations describing the relationships between the posaconazole PK parameters and their covariates are the following:

Log (Ka) = log (Ka_pop) + η_Ka;

Log (Vd/F) = log (Vd/F_pop) + η_Vd/F;

Log (CL/F) = log (CL/F_pop) + β_CL/F_age × age + η_CL/F;

where pop symbolizes the typical value of the parameter, β represents the covariate effect on the parameter, and η represents the random effect variable.

**Figure 2:**
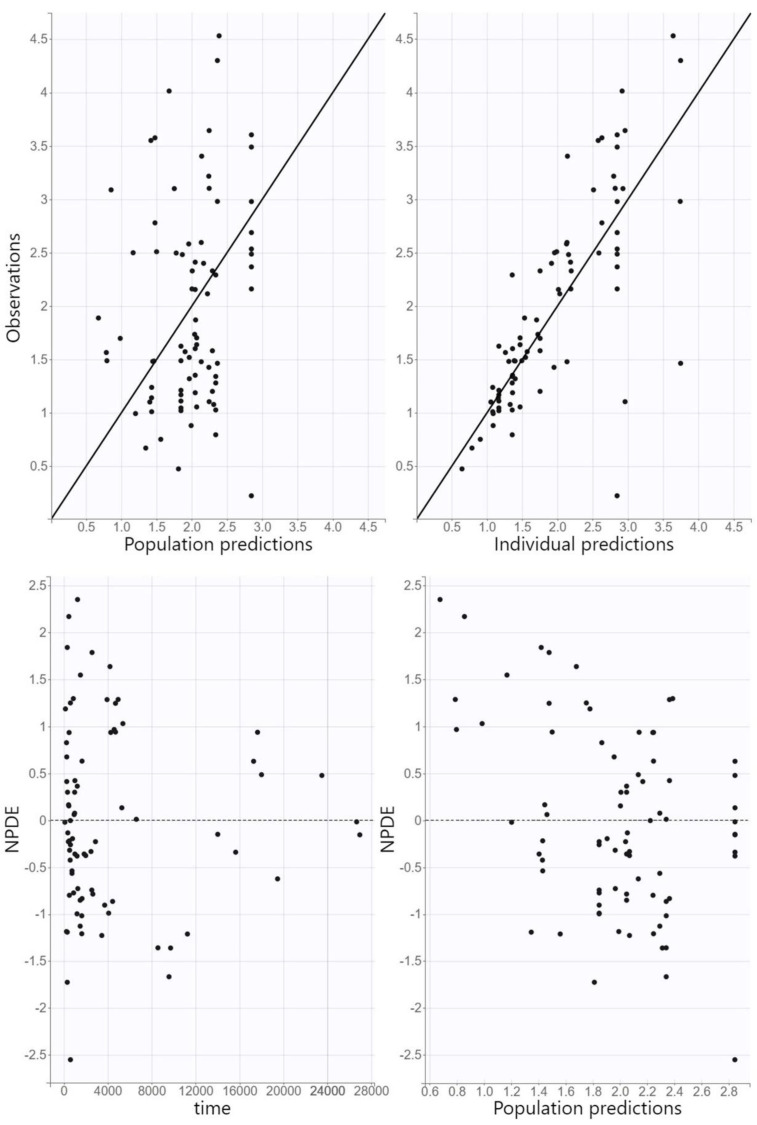
Goodness-of-fit plots: population and individual predictions against observed concentrations and normalized prediction distribution errors against time and population predictions.

**Figure 3:**
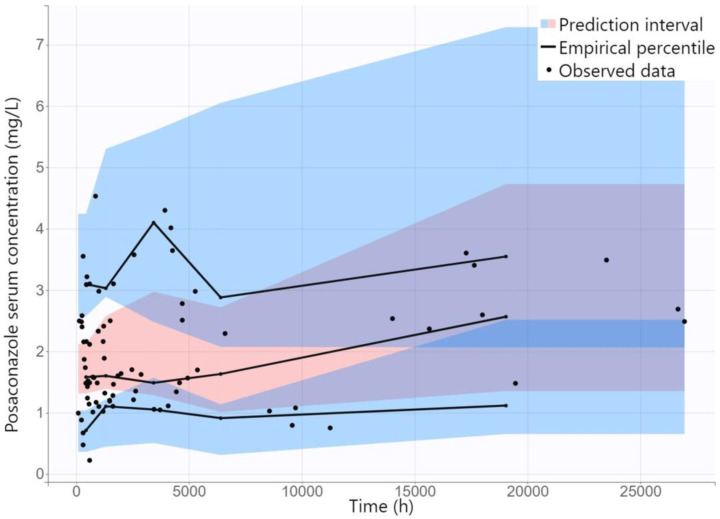
Visual predictive check of posaconazole serum concentration against time for the final model. Solid black lines represent the 10th, 50th, and 90th percentiles of the observed data. Shaded regions represent 90% confidence interval around the 10th, 50th, and 90th percentiles of the simulated data.

The diagnostic GOF plots for the final covariate model do not reveal any noticeable deviations (Figure 2). All R.S.E. values were below 50%, except β_CL/F_age, which temperately exceeded this limit, which showed that the PK parameters in the population model were relatively precisely estimated (Table 2). The VPC plot of the final model showed that the predictions were consistent with observations, confirming the validity of the PK model for the concentration–time data (Figure 3). Only wide confidence intervals that overlap indicate the high variability of posaconazole pharmacokinetics.

### 2.3. Monte Carlo Simulations

**Figure 4:**
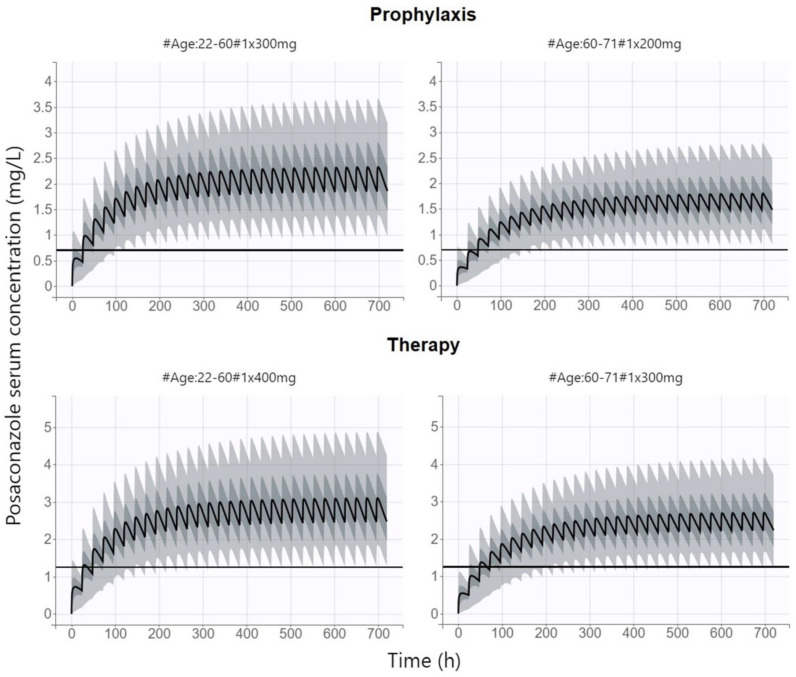
Simulated posaconazole serum concentration versus time profiles after oral administration of proposed dosing regimen of 200 mg once daily for prophylaxis in patients aged over 60 years, 300 mg once daily for prophylaxis in patients aged under 60 years or for therapy in patients aged over 60 years, and 400 mg once daily for therapy in patients aged under 60 years. The black curve represents the median, and the four grey bands represent percentiles (5–27.5%, 27.5–50%, 50–72.5%, and 72.5–95%) of the 90% simulated concentration distribution. The black line represents the posaconazole PK/PD target (trough concentration above 0.7 and 1.25 mg/L for prophylaxis and therapy, respectively).

The simulation of the theoretical distribution of posaconazole PK profiles based on a population model showed that at the approved dosage, when a uniform dose of 300 mg once daily is used in all patients for both prophylaxis and therapy, the PK/PD target recommended for prophylaxis is reached in 96% of the population, while only 81% of patients achieve the therapeutic PK/PD target. This proportion is even lower (79%) in patients under 60 years of age, in whom higher posaconazole CL/F is present. To increase the PTA, we further simulated the theoretical distribution of PK profiles at doses for prophylaxis/therapy and scaled by age as a covariate of posaconazole CL/F. The optimal prophylactic dosages of 300 mg once daily for patients under 60 years of age and 200 mg once daily for patients over 60 years of age were noted, while therapeutic doses of 400 mg and 300 mg once daily were needed in patients aged below and over 60 years, respectively. The PK/PD target for prophylaxis and therapy was met in 95% and 90% of population, respectively, representing a statistically significant increase in the PTA (*p* ˂ 0.0001) in the overall population. Moreover, the PTA in particular subpopulations was improved; the lowest PTA was observed for the therapeutic PK/PD target in the subgroup of patients over 60 years of age, but even here it was 89%. Figure 4 shows the simulated concentration–time profiles of posaconazole after oral administration of the proposed dosing regimen scaled by prophylaxis/therapy and by age.

Because the primary PK/PD index determining the efficacy of posaconazole was the AUC/MIC of 200, the probability of this target attainment was also tested. Considering that the MIC of 0.125 mg/L represents the EUCAST breakpoint for susceptible strains of *Aspergillus* spp. [13], the 24-h AUC should be at least 25 mg·h/L to meet the above-mentioned condition. At our proposed prophylaxis/therapy and age-scaled dosing, the median (IQR) values of AUC were 51 (39–66), 40 (31–51), 68 (52–89), and 60 (46–76) mg·h/L for prophylaxis in patients up to 60 years, prophylaxis in patients over 60 years, therapy in patients up to 60 years, and therapy in patients over 60 years, respectively. The overall PTA for this target was 95% in the whole population (98% for therapy and 93% for prophylaxis).

## 3. Discussion

The relatively low PK variability of posaconazole tablets in comparison with other antifungal agents and drug formulations make it an advantageous option for prophylaxis and the treatment of fungal infections. This relatively lower variability is also mirrored in the results of a retrospective study that analyzed the proportions of antifungal drug levels in the therapeutic range in lung-transplant recipients [14]. Although the posaconazole tablets showed the highest proportion of therapeutic levels (82% for therapy and 76% for prophylaxis), these results cannot be considered entirely satisfactory. Moreover, the achievement of a larger PTA for the therapeutic PK/PD target compared with the prophylactic target at identical dosing suggests a selection bias according to the enrolled population and the small number of patients in the previous study.

As there is no physiological justification for the differences in PK characteristics of the patient population for the fate of posaconazole used in therapy/prophylaxis, we analyzed the data in relation to both PK/PD goals. The posaconazole levels measured after the initiation of treatment (before the dose was adjusted based on TDM) in our study revealed that 91% of patients had levels above the prophylaxis threshold, and 72% of patients would have also met the target for therapy. The median (IQR) posaconazole serum level after the initiation of treatment was 1.61 (1.23–2.49) mg/L.

To our knowledge, this is the first study which describes the population PK of posaconazole tablets in lung-transplant recipients and proposes a covariate-based dosing optimization for both prophylaxis and therapy to improve the proportion of patients achieving the PK/PD target.

The lack of TDM data during the absorption phase did not allow us to accurately estimate the inter-individual variability of the absorption rate constant, and therefore, a fixed absorption constant of 0.8 h^−1^ was used, derived from the PK data in the SmPC. The typical value of Vd/F in our population was 386 L, which is fully consistent with other sources describing posaconazole PK in other cohorts [9,10,15]. The only identified covariate in our PK model was age for CL/F, where posaconazole CL/F of 8.8 L/h decreases by 0.009 L/h with each year of the patient´s age. This relationship is physiologically based. Many studies have reported a declining CL in various drugs in elderly patients. Although the functional capacity decreases with age mainly for phase-1 enzymes of metabolic pathways, whereas posaconazole is metabolized primarily by glucuronidation, the decreased CL may be due to the diminished hepatic blood flow and reduced liver size during aging [16]. Although age has not been identified as a covariate for CL in other posaconazole population models [10], the SmPC states that an analysis of data generated during drug development suggests that posaconazole CL is related to age [9]. None of the other characteristics tested appeared to significantly influence posaconazole PK. In some features, there is a physiological rationale for this; e.g., markers of renal function status do not predict that the elimination of a drug that is only excreted in minority by the kidneys and liver function tests is a qualitative indicator of liver damage but cannot quantify its importance for hepatic drug elimination. On the other hand, we might expect covariation in other features; e.g., cystic fibrosis is known to affect the PK of many drugs [12,17,18]. However, cystic fibrosis mainly affects the absorption phase, which could not be accurately described in our model. Furthermore, only three patients with cystic fibrosis were in our study population. On the other hand, even individual analyses of posaconazole concentrations in these patients did not suggest any trend towards higher/lower levels in comparison with other individuals. We also observed no significant changes in posaconazole dispositions during long-term therapy, which is again consistent with the previously reported low intra-patient variability [14].

We identified only one study addressing the question of whether a uniform dose of posaconazole tablets is appropriate for all patients [15]. This analysis was focused only on the use of posaconazole for prophylaxis in the cohort of patients with hematological malignancies and concluded that approximately half of the patients would benefit from a reduction of the dose from 300 mg to 200 mg. However, no covariate of posaconazole PK was found in this exploration, and therefore, the parameter according to which the dose would be individualized could not be proposed. Our analysis consistently proved that patients over 60 years of age, in whom posaconazole CL is reduced, may benefit from a dose reduction to 200 mg/day, while the recommended PK/PD target is maintained with savings of the treatment cost and a reduced risk of adverse effect occurrence. On the other hand, for the therapeutic administration of posaconazole, when we targeted for higher trough levels, we had to propose a dose increase to 400 mg/day in patients under 60 years of age to maintain the proportion of patients achieving the recommended PK/PD index.

Posaconazole is highly bound to serum proteins (˃98%), and it is generally accepted that only the unbound fraction of the drug is pharmacologically active. This would, in theory, mean that unbound concentrations of posaconazole should be below the MIC for many fungal pathogens, even at total concentrations in the therapeutic range with consequent treatment failure. However, this is contrary to the high clinical efficacy of posaconazole observed in clinical trials [11]. An in vitro study demonstrated a significant effect of posaconazole at a serum concentration of unbound posaconazole of only 10% of the MIC, whereas this effect was not observed in protein-free media. The authors suggest that the flux of protein-bound posaconazole to its fungal binding target could be an explanation for the observed effect [19].

We recognize a few limitations of our study arising from the fact that we assessed the achievement of the target PK/PD value, not the actual clinical outcomes, and also, the proposed dosage was only evaluated by simulation and has not been validated by real clinical application. It is also important to note that dose individualization does not replace TDM, but only serves to better target the initial dose; however, it is desirable that subsequent treatment be guided by the standard monitoring of serum levels. It should also be acknowledged that since the concentration data were collected only in the elimination phase (as is common in TDM practice), a fixed absorption constant had to be used, and therefore, the variability and possible covariates of this parameter could not be described. However, given that the main objective of our study was to propose individualized dosing to achieve the PK/PD target, which in the case of posaconazole is the trough level, and that CL is the most important PK parameter that determines what trough level will be achieved, we believe that our study was sufficiently powered for this aim and the dosing proposal should not be biased by a lack of sampling in the absorption phase.

## 4. Materials and Methods

### 4.1. Study Design

A prospective, observational study was conducted in adult lung-transplanted patients receiving posaconazole tablets (Noxafil, Merck Sharp & Dohme B.V., Haarlem, The Netherlands) for prophylaxis or treatment, admitted to the Third Department of Surgery of Motol University Hospital from October 2020 to March 2023. Patients meeting the following criteria were included: age ≥ 18 years, received posaconazole prophylactically or therapeutically during the postoperative period after lung transplantation, and had measured at least one serum concentration of posaconazole during the therapy. The study was authorized by the local Ethics Committee under the No. EK-873/22 on 11 August 2019 and was conducted in compliance with the Declaration of Helsinki. Written informed consent was obtained from all subjects before data collection and analysis. The study was registered in Australian New Zealand Clinical Trial Registry under the No. ACTRN12622000997752.

### 4.2. Data Acquisition

Demographic, laboratory, and clinical features of the 32 enrolled patients (10 females, 22 males) were recorded to collect information concerning gender, age, height, body weight, creatinine, alanine aminotransferase (ALT), aspartate aminotransferase (AST) and gamma-glutamyl transferase (GGT) serum levels, co-medication, and indication for lung transplantation. Body mass index (BMI), DuBois body surface area (BSA), and CKD-EPI-estimated glomerular filtration rate (eGFR) were calculated using standard formulas. Posaconazole dosing including exact time of administration was also recorded.

Posaconazole concentrations for TDM were measured in the elimination phase (exact sampling time was recorded) starting from the fourth day of treatment (expected steady state) and were repeatedly measured according to the schedule of outpatient checks throughout the period of posaconazole use (10–1123 days, median 40 days). We collected blood (5 mL) into collecting tubes with no clot activator and immediately cooled them. The samples were then centrifuged at 4500× *g* for 10 min (4 °C) and obtained serum frozen at −80 °C.

### 4.3. Bioanalytical Assay

We developed and fully validated a liquid chromatography–tandem mass spectrometry (LC-MS) method for the quantification of posaconazole in human serum with low limit of quantification of 0.1 mg/L. Unless specified elsewhere, all reagents in LC-MS quality have been obtained from Supelco, Sigma Aldrich (St. Louis, MO, USA) or Honeywell (Charlotte, NC, USA).

Agilent Technologies 1290 Infinity II LC system was used with an autosampler and 6470 Triple Quad (Agilent Technologies, Santa Clara, CA, USA). Sample separation was performed using ZORBAX SB-C8, 1.8 μm, 2.1 × 100 mm (Agilent Technologies Inc., Santa Clara, CA, USA) at 35 °C. Chromatographic separation was obtained during a total run of 6 min using gradient composition of mobile phase at a flow rate 0.5 mL/min. For the first 4 min and last 1 min of the run, a mixture starting with 70% of water (100% with 0.1% formic acid *v*/*v*) and 30% of acetonitrile (90% in water with 0.1% formic acid, *v*/*v*) was used, while 100% of acetonitrile (90% in water with 0.1% formic acid, *v*/*v*) served as a mobile phase between 4–5 min of each run.

In total, 50 µL of sample and 450 µL of internal standard (posaconazole-d4 (To-ronto Research Chemicals Inc., Toronto, ON, Canada), 0.1 mg/L dissolved in acetonitrile with water (90/10 *v*/*v*)) was used. Samples were then mixed and centrifuged for 10 min (3727 g), and 1 µL was injected into the system. The Agilent Jet Stream equipped with a heated electrospray ionization source was set in positive-ion mode. The following mass ion transitions were used for quantitation: posaconazole 701.3 *m*/*z* ^®^ 127.0 *m*/*z*, posaconazole-d4 705.4 *m*/*z* ^®^ 687.2 *m*/*z*. The bioanalytical method was validated according to the relevant EMA Guideline [20].

Linearity of the assay was proven (r^2^ = 0.9951) in the whole range (0.1–20 mg/L). The intra- and inter-day accuracy and precision in three QC samples (0.3, 1.2 and 3.5 mg/L) were determined in repeated measurements (n = 10). The intra-day and inter-day accuracies were within 2.4–3.3% and 4.0–6.8%, respectively. The intra-day and inter-day precision was within 5.5–7.9 and 2.3–8.1%, respectively.

### 4.4. Pharmacokinetic Analysis

Posaconazole concentration–time profiles were analyzed using nonlinear mixed-effects modeling (NMLE) method. The model parameters were assumed to be log-normally distributed and were estimated by maximum likelihood using the Stochastic Approximation Expectation Maximization algorithm within Monolix Suite software version 2021R2 (Lixoft SAS, Antony, France). A three-steps population PK model was developed:

(1). Base model

One- and two-compartment models with first-order absorption and elimination kinetics were tested as a structural model. PK model was parametrized in terms of absorption rate constant (K_a_), Vd, and clearance (CL). Since posaconazole was administered orally and thus the bioavailable fraction of the drug could not be estimated, the model estimates are the values of the apparent volume of distribution (Vd/F) and apparent clearance (CL/F), where F represents oral bioavailability. K_a_ was fixed to a value of 0.8 h^−1^, which was calculated from t_1/2_ and time to reach maximum plasma concentration (t_max_) values reported in SmPC according to the well-known equation t_max_ = {ln(K_a_) − ln[ln(2)/t_1/2_]}/{K_a_ − [ln(2)/t_1/2_]}. Log-normally distributed inter-individual variability terms with estimated variance were tested on Vd/F and CL/F. Additive, proportional and combined error models were tested for the residual error model. The best-fitting model was selected based on the minimum objective function value (OFV), adequacy of the goodness-of-fit plots (GOF), and low relative standard errors (R.S.E.) of the estimated pharmacokinetic parameters.

(2). Covariate model

Age, height, body weight, BMI, BSA, serum level of creatinine, ALT, AST and GGT, eGFR, and duration of posaconazole therapy were tested as continuous covariates, while gender, indication for lung transplantation, cystic fibrosis, and co-medication were tested as categorical covariates of PK parameters. Among co-medications, the concomitant treatment with cyclosporine A or tacrolimus, mycophenolate mofetil, and drugs increasing gastric pH (proton pump inhibitors or famotidine) was evaluated in a dose-independent manner [21]. A preliminary graphical assessment and univariate association using Pearson´s correlation test of the effects of covariates on estimated PK parameters was performed. The covariates with *p* < 0.05 were considered for the covariate model. A stepwise covariate modelling procedure was then performed. Assuming an χ^2^-distribution, a decrease in OFV ˃ 3.84 points between nested models (*p* < 0.05) was considered statistically significant. Additional criteria for model selection were the physiological plausibility of the obtained parameter values, absence of bias in GOF plots, and reasonably low R.S.E. values of the estimated PK parameters.

(3). Model evaluation

Model adequacy was assessed using GOF plots. Observed concentrations were plotted against individual and population predictions, while normalized prediction distribution errors (NPDE) were plotted against time and population predictions. The predictive accuracy of the final model was assessed using visual predictive check (VPC), where 1000 replicates of the original dataset were simulated using the PK parameter estimates of the final model, and the simulated distribution was compared to the distribution from the observed data. The 90% prediction intervals for the 10th, 50th, and 90th percentiles of the simulations were calculated from all replicates and presented graphically.

### 4.5. Monte Carlo Simulations

The theoretical distribution of the posaconazole PK profiles (500 replicates of all the individuals in dataset, i.e., 16,000 simulations) at various dosing regimens was generated based on final population model using Monte Carlo simulations within Simulx software version 2021R2 (Lixoft SAS, Antony, France). An approved posaconazole dosing regimen of 300 mg once daily according to SmPC was compared with the proposed covariate-scaled dosing regimen for both prophylaxis and therapy in order to increase the proportion of patients who reach the PK/PD target. Based on the EUCAST recommendation, posaconazole steady-state trough concentration ˃0.7 mg/mL for prophylaxis and ˃1.25 mg/mL for therapy was considered as the PK/PD target [13]. Alternatively, the primal PK/PD target of AUC/MIC˃200 at MIC of 0.125 mg/L (representing MIC breakpoint for susceptible strains of *Aspergillus* spp. according to EUCAST) was also tested [8,13]. Probability of target attainment (PTA) was calculated for all dosing regimens and Fisher´s exact test was used for evaluation of differences in the PTA at approved and proposed dosing regimens.

## 5. Conclusions

A population PK model of posaconazole in lung-transplant patients has been constructed based on TDM data. As the only significant covariate, age-based optimized dosing was compared with an approved uniform dose of 300 mg once daily in order to attain the PK/PD target. We proposed a dosing regimen of 200 mg once daily for prophylaxis in patients aged over 60 years, 300 mg once daily for prophylaxis in patients aged under 60 years as well as for therapy in patients aged over 60 years, and 400 mg once daily for therapy in patients aged under 60 years. At this dosing regimen, the PK/PD target for prophylaxis and therapy is met in 95% and 90% of population, respectively, representing significantly improved outcomes in comparison with those of the uniform dose.

## Figures and Tables

**Table 1:** Demographic and laboratory characteristics of patients.

	Median	IQR	Range
Age (years)	56	48–61	22–71
Height (cm)	170	170–175	151–185
Body weight (kg)	69	60–83	38–100
Body mass index (kg/m^2^)	24.4	20.7–27.0	13.1–32.4
Body surface area (m^2^)	1.81	1.68–2.00	1.40–2.20
Serum creatinine (μmol/L)	110	69–124	53–301
Estimated glomerular filtration rate (mL/s/1.73 m^2^)	0.98	0.82–1.60	0.32–2.16
Alanine aminotransferase (μkat/L)	0.72	0.42–1.03	0.24–4.97
Aspartate aminotransferase (μkat/L)	0.35	0.31–0.52	0.15–1.68
Gamma-glutamyl transferase (μkat/L)	0.63	0.34–1.31	0.20–3.30

**Table 2:** Estimates of the final posaconazole population pharmacokinetic model.

Parameter	Estimate	R.S.E. (%)
Fixed effects		
K_a__pop (h^−1^)	0.8	NA
Vd/F_pop (L)	386.35	48.1
CL/F_pop (L/h)	8.8	32.0
β_CL/F_age (L/h per year)	−0.009	63.0
Standard deviation of the random effects		
Ω_K_a_	3.43	27.5
Ω_Vd/F	0.45	47.7
Ω_CL/F	0.36	20.3
Error model parameters		
Proportional	0.29	11.3

K_a_ is absorption rate constant after oral administration. Vd/F is apparent volume of distribution. CL/F is apparent clearance.

**Table 3:** *p*-values for relationships of potential covariates tested and posaconazole PK parameters.

Potential Covariate Tested	K_a_	Vd/F	CL/F
Gender	0.810	0.141	0.700
Indication for lung transplantation	0.167	0.821	0.600
Cystic fibrosis	0.978	0.715	0.769
Calcineurin inhibitor	0.220	0.987	0.470
Mycophenolate mofetil	0.496	0.496	0.096
Drugs increasing gastric pH	0.078	0.479	0.322
Age	0.174	0.915	0.012 *
Height	0.842	0.889	0.663
Body weight	0.647	0.491	0.426
Body mass index	0.679	0.410	0.277
Body surface area	0.677	0.557	0.608
Serum creatinine	0.501	0.432	0.723
eGFR	0.172	0.989	0.932
ALT	0.676	0.335	0.224
AST	0.818	0.151	0.965
GGT	0.863	0.989	0.932

* significantly associated. K_a_ is absorption rate constant after oral administration. Vd/F is apparent volume of distribution. CL/F is apparent clearance.

## Data Availability

The data that support the findings of this study are available from the corresponding author upon reasonable request.
